# Case Report: Diffuse T wave inversions as initial electrocardiographic evidence in acute pulmonary embolism

**DOI:** 10.12688/f1000research.14927.2

**Published:** 2018-09-06

**Authors:** Ogechukwu Egini, Alix Dufresne, Mazin Khalid, Chinedu Egini, Eric Jaffe

**Affiliations:** 1 Department of Medicine, Interfaith Medical Center, Brooklyn, NY, 11213, USA; 2 Division of Cardiology, Interfaith Medical Center, Brooklyn, NY, 11213, USA; 3 University of Port Harcourt, Port Harcourt, Nigeria; 4 Internal Medicine Residency Program, Interfaith Medical Center, Brooklyn, NY, 11213, USA

**Keywords:** PE, T-waves, inversion

## Abstract

Acute pulmonary embolism (PE) is a life-threatening condition and is typically diagnosed by a combination of symptoms, clinical signs and imaging. Electrocardiogram may be helpful in diagnosis, and the most widely described pattern of occurrence is the so-called S _1_Q _3_T _3_ pattern. Here, we describe the case of an African-American male who presented with typical chest pain, diffuse T wave inversions with serial troponin elevation. There was initial concern for Wellen's syndrome but was finally diagnosed as acute PE. This case underscores the necessity of vigilance and a lower threshold for PE work up even in patients presenting as acute coronary syndrome.

## Introduction

Acute pulmonary embolism (PE) is responsible for 20–25% of sudden death in the United States ^1, 2^. It exacts a huge economic burden both on the sufferer and the health system. In their study to assess PE and deep vein thrombosis (DVT) inpatient costs in the United States, Lamori *et al*. found that the mean cost of initial hospitalization for acute PE was approximately $37,006 per patient ^3^. This figure was higher for older patients, women and readmissions. Prompt diagnosis is, therefore, essential to reduce disease burden. The so-called S _1_Q _3_T _3_ pattern is the classic electrocardiogram (EKG) presentation in acute PE ^4^ but is not seen in all acute PE cases. We present the case of acute PE with initial clinical presentation that mimicked acute coronary syndrome and an initial EKG pattern that suggested Wellen’s syndrome.

## Case report

A 66 year old African-American male presented to the Emergency Room (ER) complaining of a 2-hour history of chest pain. Chest pain was described as left-sided, non-pleuritic, non-radiating, retrosternal, squeezing in character and persistent. Pain was reported as 9 on a 10-point pain scale and relieved by taking 0.4mg tablet of nitroglycerin sublingually. It was associated with shortness of breath, dizziness and sweating, but the patient denied loss of consciousness, cough, palpitation or swelling of the extremities. He denied any use of illicit substances. A week prior to this hospitalization he presented to the hospital with a similar complaint. At that time, chest pain was relieved by 325mg dose Aspirin taken orally; troponin was normal and EKG did not show any significant change from baseline. His echocardiogram was also normal and he was discharged with scheduled outpatient stress test. Medical history was significant for poorly-controlled diabetes type 2, hypertension, dyslipidemia and obesity.

On this visit, his pulse rate was 84 beats per minute; BP 119/66 mm/Hg; respiration rate 16 breaths per minute and his oxygen saturation was 98% on room air. Initial troponin was elevated at 0.19ng/ml (reference 0.00 – 0.05ng/ml); hemoglobin of 14.4g/dl (reference 13–17g/dl) and platelet count of 210 × 10 ^3^/ul (reference 130–400 × 10 ^3^/ul).

**Figure 1.**
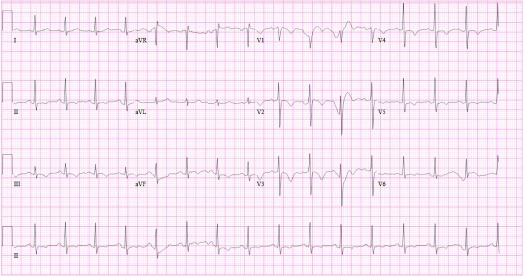
EKG showing deep T wave inversions in leads V1–V6 and the inferior limb leads.

**Figure 2.**
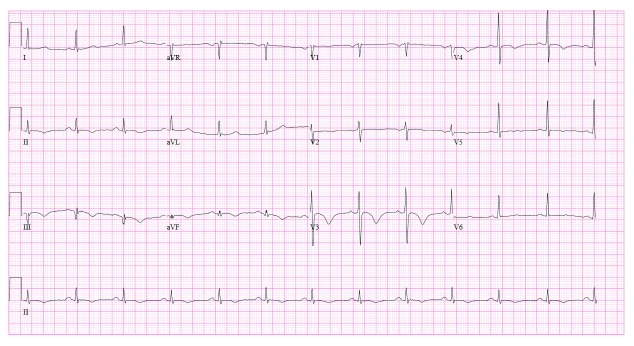
Repeat EKG now showing a Q _3_T _3_ pattern in lead III.

**Figure 3.**
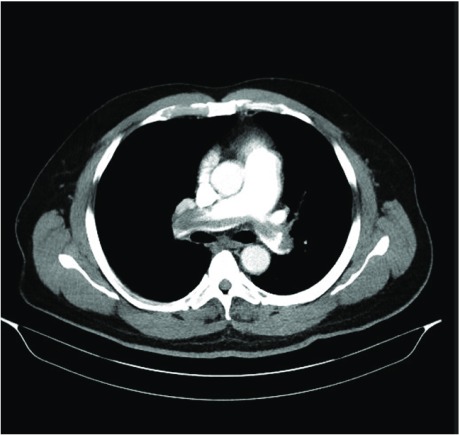
Axial CTA of the chest showing a saddle embolus with extension into the branches of the pulmonary artery. CTA was performed using Siemens SOMATOM Perspective 128 slices. Images were obtained in a cranio-caudal direction following contrast injection at 3mls/s. Contrast optimization was based on bolus tracking at the level of the main pulmonary artery using a trigger level of 100 HU.

EKG showed deep T wave inversions in leads V1–V6 and the inferior limb leads ( Figure 1). We assumed an assessment of non-ST elevation myocardial infarction and a loading dose of Aspirin (325 mg) and Plavix (300 mg) were given orally in the ER along with Atorvastatin (80 mg) and a weight-based dose of Enoxaparin. Repeat troponin 6 hours later was 1.05. Cardiac catheterization revealed normal coronaries ( Supplementary file 1). While the patient was still lying on the cardiac cath table, his oxygen saturation dropped to 91%. There was no chest pain, tachypnea or tachycardia at this time. Supplemental oxygen at 2l/min via nasal cannula improved saturation to 97%. A repeat EKG showed a Q _3_T _3_ pattern in lead III ( Figure 2). In view of these new findings (low oxygen saturation and a change in the EKG pattern), a computerized tomography of the chest with angiogram (chest CTA) was ordered. This revealed a saddle pulmonary embolus which extended into the right and left pulmonary arteries and involved all lobar branches of the pulmonary arteries ( Figure 3).

Treatment was continued with Enoxaparin (100mg subcutaneously every 12 hours) for 6 days, at which time he became stable and maintained oxygen saturation above 96% even when supine. He was discharged on Apixaban (10mg po bid for 7 days followed by 5mg po bid) with plan to complete 3 months of therapy. Follow up visits were scheduled with the Cardiology and Hematology clinics.

## Discussion

Acute pulmonary embolism (PE) is caused by blockage of a pulmonary artery by blood clot. In one study, investigators found that the commonest clinical symptoms in acute PE patients were dyspnea, chest pain, syncope and hemoptysis ^4^. A number of EKG findings have been described in acute PE patients but the classic EKG finding is the S _1_Q _3_T _3_ pattern ^5^. The incidence of this pattern in acute PE is highly variable ^5^. Other EKG changes have been reported in patients diagnosed with PE ^6^ but there were initial supporting clinical evidence to warrant suspicion and further diagnostic testing for PE. On the contrary, our patient presented with features suggestive of acute coronary syndrome - typical chest pain, diffuse T wave inversions and elevated cardiac enzymes. Pulse rate, respiration rate and oxygen saturation were normal essentially making an acute PE assessment difficult at time of presentation. Given a background of significant cardiovascular risk factors, a coronary event was thought more likely. Deep T wave inversions on the precordial leads were concerning for Wellen’s syndrome ^7^. The clues to possible acute PE in our case was the transient desaturation that occurred during cardiac catheterization and the observed change on repeat EKG. These dictated the urgency of getting a chest CTA. The chest CTA is the gold standard for diagnosis of PE and was shown in the Prospective Investigation of Pulmonary Embolism Diagnosis II (PIOPED II) to have a high sensitivity and specificity for acute PE diagnosis and was also concordant with the pretest Well’s criteria ^8^. A ventilation-perfusion (V/Q) scan may also effectively diagnose acute PE and is useful in renal insufficiency or contrast allergy. Treatment of acute PE is based on risk stratification. Anticoagulation is the mainstay of therapy and the duration of treatment is determined by a number of factors including provoked vs unprovoked PE and/or recurrence of acute PE. Those with acute PE and hypotension without significant bleeding risk require thrombolysis ^9^. In some cases of massive PE with contraindication to or failure of systemic fibrinolysis, surgical or catheter embolectomy can be considered ^10^.

## Conclusion

Acute pulmonary embolism should be considered as a differential in patients with deep T wave inversions on EKG who do not have typical PE presentation.

## Consent

Written informed consent for the publication of the patient’s clinical details and clinical images was obtained from the patient.

## Data availability

All data underlying the results are available as part of the article and no additional source data are required.

## Supplementary material

**Supplementary File 1.** Video file of angiogram showing patent coronaries
