# Supplementary material for: Case Report: Diffuse T wave inversions as initial electrocardiographic evidence in acute pulmonary embolism
Source: F1000Res. 2018 Sep 6;7:738. Originally published 2018 Jun 13. [Version 2] doi: 10.12688/f1000research.14927.2 (PMC6178910; doi:10.12688/f1000research.14927.2)
Supplement: Peer review history

# Peer review: Referee response for version 2

**DOI:** 10.5256/f1000research.17710.r38032

**Published:** 2018-10-05

**Authors:** Chukwudi Obiagwu

**Affiliations:** 1 Department of Cardiology, Maimonides Medical Center, New York City, NJ, USA

I thank the authors for reviewing the manuscript. Interesting report.

I have read this submission. I believe that I have an appropriate level of expertise to confirm that it is of an acceptable scientific standard.

# Peer review: Referee response for version 1

**DOI:** 10.5256/f1000research.16252.r35031

**Published:** 2018-07-10

**Authors:** Chukwudi Obiagwu

**Affiliations:** 1 Department of Cardiology, Maimonides Medical Center, New York City, NJ, USA

The author describe a case of acute PE initially masquerading as acute MI. The authors do a good job presenting a manuscript that is easy to read. However, there are a few points to be noted:

1. Introduction:
  The authors on citing the cost of care for PE rely on an old article whereas there are more recent articles providing more current cost estimates. See citation added below ^1^. 
2. The authors use of punctuation marks while describing the characteristics of chest pain needs to be reviewed.
3. Is "in this visit" the right phrase to use or "on this visit" the proper one?
4. A review of this sentence "Oxygen saturation dropped to 91% in room air while the patient laid on catheterization table but improved with supplemental oxygen via nasal cannula" is needed.
5. The authors would do well to provide an image of the coronary angiogram and CTA PE protocol.
6. Post coronary angiogram, while in the cath lab, did the patient still complain of chest pain, was he tachypneic, or in distress that led to desaturation to 91%? A lot health care providers will not get a CTA chest for O2 saturation of 91% without other symptoms.
  On seeing normal coronary arteries, prior symptoms could have been ascribed to MINOCA. See citation 2 ^2^.

I have read this submission. I believe that I have an appropriate level of expertise to confirm that it is of an acceptable scientific standard, however I have significant reservations, as outlined above.

## References

[1] LaMoriJCShoheiberOModySHBookhartBK: Inpatient resource use and cost burden of deep vein thrombosis and pulmonary embolism in the United States. *Clin Ther*.2015;37(1) : 10.1016/j.clinthera.2014.10.024 62-70 10.1016/j.clinthera.2014.10.024 25524389

[2] PasupathySAirTDreyerRPTavellaRBeltrameJF: Systematic review of patients presenting with suspected myocardial infarction and nonobstructive coronary arteries. *Circulation*.2015;131(10) : 10.1161/CIRCULATIONAHA.114.011201 861-70 10.1161/CIRCULATIONAHA.114.011201 25587100

# Response

**Published:** 2018-09-04

**Authors:** Ogechukwu Egini

**Affiliations:** Interfaith Medical Center, USA

Thank you for the responses.

We have updated Version 2 to reflect more recent analysis of the cost imperative of treating acute PE in a US hospital and have also included images of both coronary angiogram and an axial cut of the CTA PE. MINOCA is a consideration in this situation. However, given the 2 new findings of a drop in oxygen saturation and the change in EKG pattern, we thought it was best to rule out acute PE at that time.

# Peer review: Referee response for version 1

**DOI:** 10.5256/f1000research.16252.r35034

**Published:** 2018-07-03

**Authors:** Chibundo Uchenna Nwaneli

**Affiliations:** 1 Department of Medicine, Faculty of Medicine, Nnamdi Azikiwe University, Awka, Nigeria

The article I reviewed is a case report of 66 year old African American who presented to the hospital with recurrent chest pain whose initial evaluation was thought to be consistent with coronary artery disease. He was subsequently found to have Acute pulmonary embolism on Chest CT angiogram and normal coronary vessels. This case is quite intriguing because of the  presentation. It throws up some question I would like the authors to try to answer and offer explanation.

1. The initial presentation with chest pain which resolved with administration of 325mg aspirin: did the team think it was pulmonary embolism.Is it usual for embolism to respond with single low dose aspirin.
2. The relief of patient's pain with Nitroglycerin, is it typical with chest pain from embolism.
3. Is it possible that we are dealing with Non ST segment myocardial infarction (NSTEMI) coexisting with pulmonary embolism.
4. Myocardial infarction occurring in the setting of normal coronary arteries have been reported. Could this be the case here knowing fully well that this patient had multiple risk factors for coronary artery disease.

The EKG findings observed in the index patient have been described in pulmonary embolism as well as the elevated troponin. They could as well be found in NSTEMI. 

 Treatment for submassive pulmonale embolism and NSTEMI  both require anticoagulation.

 I dont know how many pictorials that are allowed for case report by the journal but I would like the authors to include the baseline EKG of the patient on his first presentation to the hospital. If also permissible the Chest CT and Coronary angiogram images.This I think will enable readers agree with their conclusions.   

 Overall the case report was well written.  

 The take home message is that diagnosis of pulmonary embolism can be difficult especially when the features mimic other conditions such as acute coronary syndrome. EKG changes may give a clue to the diagnosis. As clinicians we know the inexactness of scientific data and we should  have an open mind to other differential diagnosis.

I have read this submission. I believe that I have an appropriate level of expertise to confirm that it is of an acceptable scientific standard.
